# Preliminary Studies of Antimicrobial Activity of New Synthesized Hybrids of 2-Thiohydantoin and 2-Quinolone Derivatives Activated with Blue Light

**DOI:** 10.3390/molecules27031069

**Published:** 2022-02-05

**Authors:** Agnieszka Kania, Waldemar Tejchman, Anna M. Pawlak, Krystian Mokrzyński, Bartosz Różanowski, Bogdan M. Musielak, Magdalena Greczek-Stachura

**Affiliations:** 1 Institute of Biology, Pedagogical University of Krakow, Podchorążych 2, 30-084 Kraków, Poland; waldemar.tejchman@up.krakow.pl (W.T.); bartosz.rozanowski@up.krakow.pl (B.R.); magdalena.greczek-stachura@up.krakow.pl (M.G.-S.); 2 Department of Biophysics, Faculty of Biochemistry, Biophysics and Biotechnology, Jagiellonian University, Gronostajowa 7, 30-387 Kraków, Poland; anna.pawlak@uj.edu.pl (A.M.P.); mokrzynskikrystian@gmail.com (K.M.); 3 Department of Organic Chemistry, Faculty of Chemistry, Jagiellonian University, Gronostajowa 2, 30-387 Kraków, Poland; musielak@chemia.uj.edu.pl

**Keywords:** hybrid of 2-thiohydantoin and 2-quinolone derivative, blue light activation, singlet oxygen generation, antibacterial test activity

## Abstract

Thiohydantoin and quinolone derivatives have attracted researchers’ attention because of a broad spectrum of their medical applications. The aim of our research was to synthesize and analyze the antimicrobial properties of novel 2-thiohydantoin and 2-quinolone derivatives. For this purpose, two series of hybrid compounds were synthesized. Both series consisted of 2-thiohydantoin core and 2-quinolone derivative ring, however one of them was enriched with an acetic acid group at N3 atom in 2-thiohydantoin core. Antibacterial properties of these compounds were examined against bacteria: *Staphylococcus aureus*, *Bacillus subtilis*, *Enterococcus faecalis*, *Escherichia coli*, *Pseudomonas aeruginosa*, and *Klebsiella pneumoniae*. The antimicrobial assay was carried out using a serial dilution method to obtain the MIC. The influence of blue light irradiation on the tested compounds was investigated. The relative yield of singlet oxygen (^1^O_2_*, ^1^Δ_g_) generation upon excitation with 420 nm was determined by a comparative method, employing perinaphthenone (PN) as a standard. Antimicrobial properties were also investigated after blue light irradiation of the suspensions of the hybrids and bacteria placed in microtitrate plates. Preliminary results confirmed that some of the hybrid compounds showed bacteriostatic activity to the reference Gram-positive bacterial strains and a few of them were bacteriostatic towards Gram-negative bacteria, as well. Blue light activation enhanced bacteriostatic effect of the tested compounds.

## 1. Introduction

Antibacterial resistance of pathogenic microorganisms is a global problem in the health care, agriculture, and food industries. Therefore, many scientific groups are constantly in search for novel compounds demonstrating an antimicrobial potential. Among already applied drugs, quinolones and thiohydantoin derivatives are used as chemotherapeutics and antibiotics. Compounds containing 2-thiohydantoin core reveal antibacterial [1,2,3], antifungal [4], antitumor [5], antiparasitic [6], and antiviral activity [7]. Compounds with 2-quinolone core are also known for their broad range of biological effects [8,9]. They possess anticancer [10,11], antituberculosis [12], antibacterial [13], and antifungal properties [14]. Many of the tested compounds are hybrids of 2-quinolone derivatives and other heterocyclic systems [15]. 2-quinolone and benzo[d]thiazoyl hybrids reveal not only antitumor activity but also reduce the growth of Gram-negative bacteria [16]. 2-quinolone and 1,2,4-triazole hybrids and 2-quinolone and coumarin hybrids also show activity against Gram-negative bacteria [17]. An interesting antimalarial activity is demonstrated by 2-quinoline and pyrimidine-2-thione hybrids containing an aryl group at the nitrogen atom of the quinolone system [18]. Because of a narrow antimicrobial spectrum of quinolones, covering Gram-negative bacteria and a few Gram-positive ones, various chemical modifications of quinolones, such as fluorine substituent introduction, have increased its antibacterial spectrum [19]. Fluoroquinolones possess excellent activity against bacteria of the family *Enterobacteriaceae* and other Gram-negative bacteria, such as *Haemophilus influenzae*, *Neisseria gonorrhoeae*, *Neisseria meningitidis*, and *Moraxella catarrhalis* [20]. 

Various classes of photoactive chemical compounds, such as porphyrins and phenothiazines, have been described as photoinactivating agents against Gram-positive and Gram-negative bacteria [21,22,23,24,25]. After absorption of light by a photosensitizer molecule, part of the energy is transferred to its triplet state. The excitation energy of the triplet state is transferred to a substrate (Type I reaction) or to molecular oxygen (Type II reaction) to generate reactive oxygen species (ROS) that cause further oxidative reactions [26]. Therefore, the photodynamic effect directed to bacteria cell wall, lipid membranes, enzymes or nucleic acid damages bacteria cells after irradiation [27]. Proteins are considered to be the main target of ROS. As it was reported by Michaeli et al. [28], singlet oxygen reacts with aromatic amino acids and with those containing sulfur, which results in the accumulation of toxic products. In DNA ROS may lead to frameshift mutation [29]. The cell membrane integrity and active transport processes are disturbed due to lipid peroxidation. The bactericidal effects of blue light radiation have been studied in many bacterial strains, such as *Propionibacterium acnes*, *Helicobacter pylori*, *Escherichia coli*, *Staphylococcus aureus*, and *Pseudomonas aeruginosa* [30,31,32,33]. Gram-positive and Gram-negative bacteria vary significantly in response to ROS sensitive molecules, because of the structural differences of the cell envelopes [34].

In our previous work [2], we compared the activity of 2-thiohydantoin and 2-thiohydantoin-3-acetic acid derivatives, containing bulky substituents at C5 position, dependently on the size of these substituents. We stated that the introduction of such bulky substituents led to the decrease of the antibacterial activity. Therefore, we decided to apply other smaller moieties and to introduce electron withdrawing and electron donating groups into them. As a model system, we chose 2-quinolones. 

We synthesized two series of novel hybrids of 2-thiohydantoin and 2-quinolone derivatives, expecting a synergistic effect of these two heterocyclic systems on the bacteria growth inhibition. Both series consisted of a 2-thiohydantoin core and 2-quinolone ring; however, the one of them was enriched with an acetic acid group at N3 atom in 2-thiohydantoin ring. Based on literature reports, we presumed that these compounds might have a broad spectrum of activity, specially towards Gram-positive bacteria. To increase the antibacterial effect of the hybrids, blue light irradiation was applied. Generated singlet oxygen should enhance a bacterial growth inhibition. The main aim of our studies was the chemical synthesis and modifications of the novel hybrid derivatives, their spectroscopic and preliminary photophysical characterization as well as preliminary confirmation of their biological activity in vitro towards the selected bacterial strains.

## 2. Results and Discussion

### 2.1. Chemistry

**Scheme 1:**
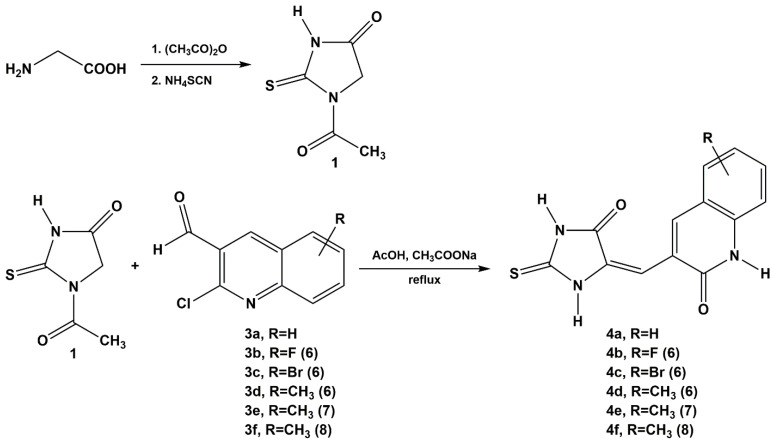
Synthesis of 1-acetyl-2-thiohydantoin and its condensation with 2-chloroquinoline-3-carbaldehyde derivatives.

The Knoevenagel condensation of 1-acetyl-2-sulfanylideneimidazolidin-4-one (the common name: 1-acetyl-2-thiohydantoin) with 2-chloroquinoline-3-carbaldehyde derivatives was carried out according to the modified procedure described earlier [35]. The reaction was conducted in acetic acid solution in the presence of sodium acetate by heating the reaction mixture under reflux within 5 to 6 h (Scheme 1). Under the conditions used, the acyl group was cleaved from 1-acetyl-2-thiohydantoin. Due to this, the final product obtained as a result of condensation contains 2-thiohydantoin core [36].

**Scheme 2:**
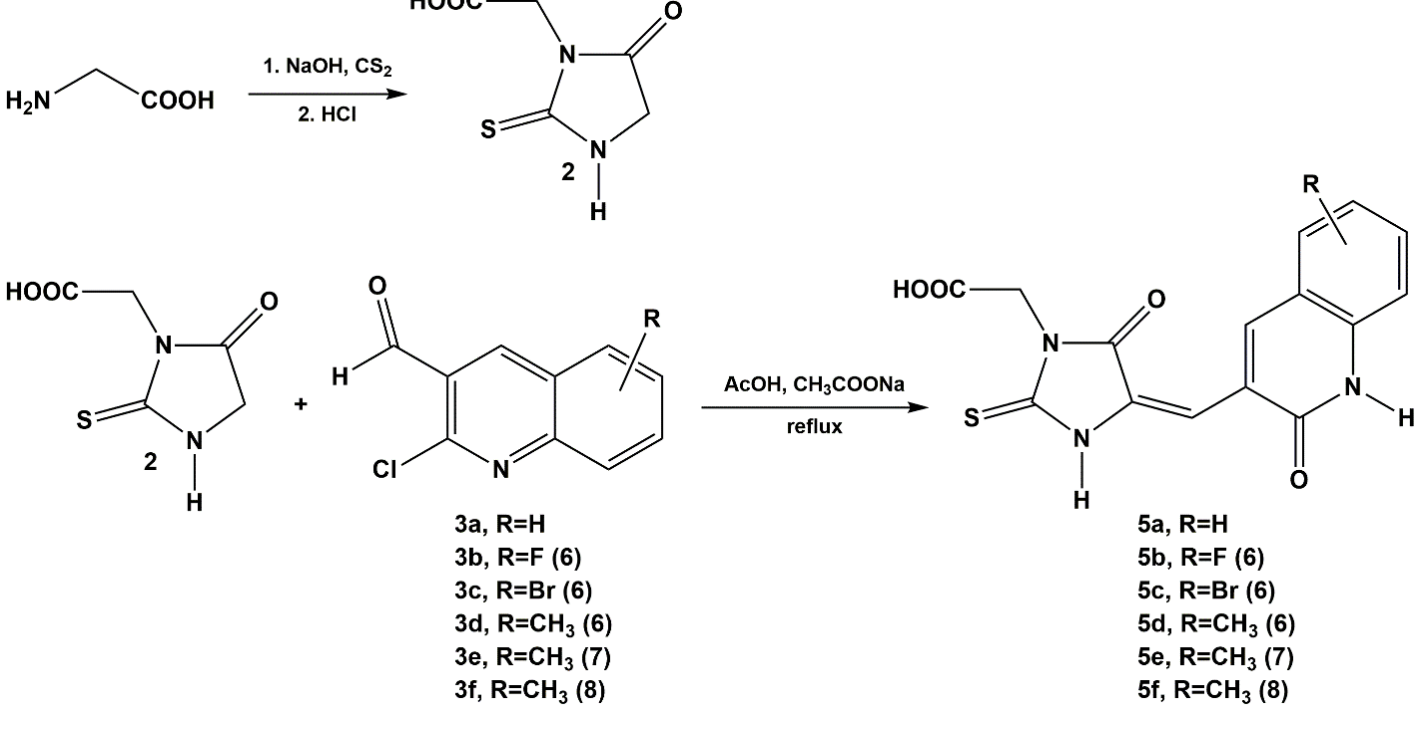
Synthesis of 2-thiohydantoin-3-acetic acid its condensation with 2-chloro-3-quinolinecarboxaldehyde derivatives.

The condensation procedure of 2-(4-oxo-2-sulfanylideneimidazolidin-3-yl) acetic acid (the common name: 2-thiohydantoin-3-acetic acid) was carried out analogously with 2-chloroquinoline-3-carbaldehyde derivatives (Scheme 2).

In both series of syntheses, the chlorine atom in C-2 position was replaced with an oxygen atom, which led to the preparation of derivatives containing the 2-quinolone core. The obtained products were the result of the applied reaction conditions. The reactions were carried out in glacial acetic acid and in the presence of sodium acetate. Previously, Selvi and Nadaraj [37] observed the transformation of 2-chloroquinoline-3-carbaldehyde into 2-oxo-1,2-dihydroquinoline-3-carbaldehyde in such a reaction environment (the milieu of acetic acid and sodium acetate) under the influence of microwave radiation. It seems that the presence of acetic acid was of decisive importance in this case since the reactions taking place with the participation of 2-chloroquinoline derivatives in ethanol medium and in the presence of piperidine did not lead to 2-oxo-1,2-dihydroquinoline derivatives [17]. The obtained compounds were characterized by very low solubility in popular solvents. In DMSO, which we used as a basic solvent in the biological part, two of the compounds, 5a and 5d, were not soluble. During the determination of the melting points, they did not melt but decomposed at a temperature of approx 350 °C. 

In the recorded absorption spectra of the investigated compounds there are several bands observed in the wavelength range 340–510 nm, with the main two ones around 415–420 nm and 440 nm (DMSO: ethanol (1:1, *v*/*v*)) (see Figure S1 in Supplementary Materials). In the emission spectra recorded in the same solvent system there appears the main band with maximum placed at around 480 nm (see Figure S2 in Supplementary Materials).

### 2.2. Direct Detection of Singlet Oxygen (^1^Δ_g_, ^1^O_2_*) Phosphorescence at 1270 nm

**Figure 1:**
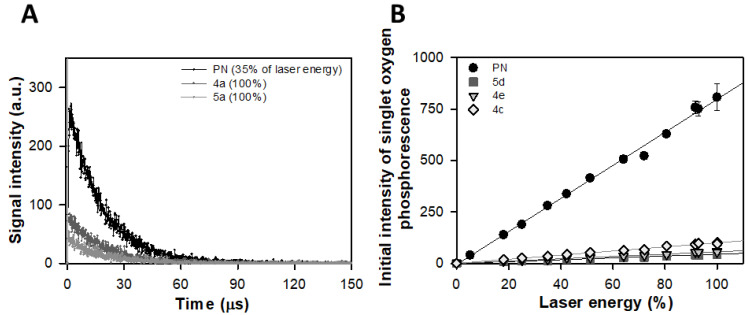
(**A**) Time-resolved singlet oxygen (^1^O_2_, ^1^Δ_g_) phosphorescence at 1270 nm detected in DMSO: ethanol solutions (1:1, *v*/*v*) of perinaphtenone (PN) and selected samples (**4a** and **5a**) upon excitation with 420 nm laser pulses at 35% and 100% of laser energy, respectively. (**B**) Dependence of the initial intensity of ^1^O_2_ (^1^Δ_g_) phosphorescence generated by PN and selected samples on relative excitation energy.

All studied quinolone derivatives generate singlet oxygen upon excitation with 420 nm, however with different efficiency. Decays of characteristic ^1^O_2_ phosphorescence generated by selected samples and PN in DMSO: ethanol (1:1, *v*/*v*) solution equilibrated with air upon excitation with laser pulse are presented in Figure 1. 

**Figure 2:**
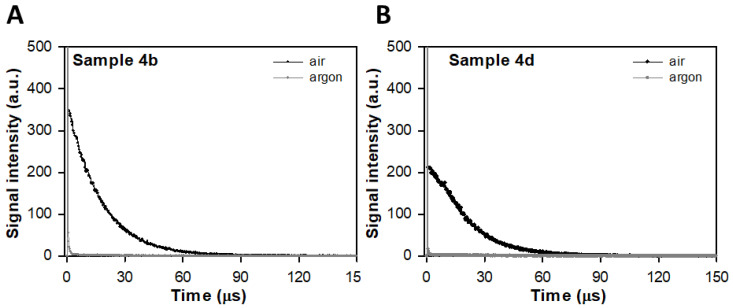
Singlet oxygen (^1^O_2_, ^1^Δ_g_) phosphorescence decay detected at 1270 nm after laser excitation of selected samples: **4b** (**A**) and **4d** (**B**) in DMSO: ethanol solution (1:1, *v*/*v*) with 420 nm. Studied samples solutions were equilibrated with air (black) or saturated with argon (grey).

The observed phosphorescence of ^1^O_2_, generated by PN and quinolone derivatives, decayed with time constant 17.50 (±0.27) μs and 17.36 (±0.20) μs, respectively. Observed singlet oxygen lifetime (τ_Δ_) in a mixture of solvent used has not been reported previously, however it was expected that resultant τ_Δ_ would differ from that recorded in each solvent separately [38,39]. After saturation of studied samples solutions with argon, singlet oxygen luminescence was not observed (Figure 2).

PN belongs to a class of aromatic ketones, with well-known singlet oxygen photosensibilization properties. PN possesses many desirable attributes for use as a standard in ^1^O_2_ (^1^Δ_g_) generation efficiency determination studies using comparative method [40]. Quantum yield (Φ_Δ_) of singlet oxygen generation by PN reaches value of 1.00 in acetonitrile, 0.96 in dichloromethane, 0.92 in toluene and ethanol, and 0.91 in cyclohexane [41]. According to our best knowledge, there is no data on Φ_Δ_ by PN in solvents mixture used in our studies. It is why we cannot provide values of absolute quantum yield of ^1^O_2_ generation by studied samples. Relative efficiency of ^1^O_2_ (^1^Δ_g_) generation by quinolone derivatives studied in this work, determined in DMSO: ethanol (1:1, *v*/*v*), are between 12 and 3% of the efficiency observed for PN (Table 1, Figure 2). However, as PN is a very effective ^1^O_2_ sensitizer in a wide range of solvents, so it can be expected that Φ_Δ_ of singlet oxygen generation in DMSO: ethanol mixture is rather high and not lower than 0.9 [42].

Quinolones, as most of the drugs in this group, reveal phototoxicity [43]. There is a correlation between phototoxicity and the drug structure. Halogenated quinolones, especially those with F in the position 8 (fluoroquinolones, FLQ) seem to be more phototoxic. They generate singlet oxygen and superoxide anion, however determined quantum yields of ^1^O_2_ (^1^Δ_g_) generation for selected FLQ were rather low ranging from 0.06 to 0.09 [43]. These values are quite close to that observed by us for other quinolones derivatives. It has also been shown that naphthyl esters derivatives of quinolones generate singlet oxygen under irradiation with visible light that combined with their good hydrophilicity make them promising candidates as PDT agents [44,45].

### 2.3. Antibacterial Activity

#### 2.3.1. Antibacterial Activity under Dark Conditions

The preliminary antimicrobial activity of novel synthesized hybrid compounds was assayed in the context of creating new drug-like molecules. In the present study, the antimicrobial assay of these compounds was carried out towards reference bacterial strains using a serial dilution method to obtain the MIC and MBC values (Table 2, Table 3 and Table 4). Some of the compounds were neither bactericidal nor bacteriostatic what is assigned in the tables as ‘not determined’ (nd). In dark conditions most of the tested derivatives had activity against Gram-positive bacteria (*S. aureus*, *B. subtilis*, and *E. faecalis*) and/or Gram-negative bacteria (*E. coli*, *K. pneumoniae*, *P. aeruginosa*) (Table 2 and Table 3). Each compound affected at least one bacterial strain, apart from **5e** and **4a**. **4b** and **5c** were active towards *E. faecalis* and *S. aureus* (**5c** also towards *B. subtilis*). **4e** inhibited the growth of all the bacteria strains, however much more strongly in case of Gram-positive bacteria. **5f** and **4f** inhibited the growth of all the bacterial strains, whereas **4d** all the Gram-positive bacteria and *K. pneumoniae*. Both compounds, **5f** and **4f**, turned out to be antibacterial active, however **4f** possessing no acetic group at N3 in 2-thiohydantoin ring is much more potent in comparison to **5f** having this group. Similarly, a lack of growth inhibition effect was observed in case for **5e** with acetic group at N3 in comparison to **4e** differing only with this group. **5d** and **5a** were not soluble in DMSO, therefore the antibacterial tests were not performed for those two compounds. Concluding, we observed the in vitro bacteriostatic effect of Gram-positive and Gram-negative bacteria to the novel 2-thiohydantoin and 2-quinolone derivatives hybrids. The growth inhibition was noticed in case of all the tested bacterial strains or only Gram-positive ones, dependently on the compound used. However, this bacteriostatic effect was never observed exclusively for Gram-negative bacteria.

All tested bacterial strains were sensitive to ciprofloxacine and the MIC values were 0.24–7.81 μg/mL. The greatest sensitivity was observed in case of Gram-negative bacteria: *E. coli* and *P. aeruginosa* (Table 5). On the other hand, our compounds were mild or moderate active towards Gram-positive bacteria. However, they did not exhibit the bactericidal activity. Table 4 shows that the tested compounds (**4b**, **4d**, **4e**, **4f**, **5b**, **5c**, **5f**) had a weak to moderate bacteriostatic activity. In contrast, they had no bactericidal activity as indicated by MBC/MIC ratios that equal 8 or more (8–64).

The major mechanism of quinolone resistance is known to be an alteration of the target enzyme DNA gyrase (topoisomerase II) or topoisomerase IV and rapid inhibition of bacterial DNA synthesis leads to cell death [19,46,47,48].

Most of the synthesized compounds exhibited moderate or mild bacteriostatic activity amongst the tested Gram-positive bacteria. The cell wall of Gram-positive bacteria consists of peptidoglycan whereas in Gram-negative bacteria a thin layer of peptidoglycan constitutes only 10% of the cell wall. The outer bilayer phospholipid membrane contains lipopolysaccharide (LPS), porin, and adhesin proteins. In case of *E. coli* belonging to Gram-negative bacteria, the outer membrane contains porin proteins OmpF and OmpC, which allow essential solutes and other molecules enter the cell. Downregulation of these porins can lead to reduced accumulation of quinolones (and other agents) within the cell [49,50]. On the other hand, Gram-positive bacteria active efflux transporters are powered by the proton gradient across the cytoplasmic membrane, being the principal means of reducing cytoplasmic drug concentrations [46]. Based on literature reports, we suspect that the synthetic agents such as our hybrids may be a group of substrates for efflux pumps. Besides, transport into the cell is connected with the size of the molecule—e.g., a quinolone molecule chelated with Ca^2+^ or Mg^2+^. Such a complex molecule cannot enter the cell because of the bulky size [51]. The effect of greater sensitivity of Gram-positive bacteria to the examined compounds is also consisted with those reported for other 2-thiohydantoins derivatives. Trotsko et al. [52] described similar effect for new hybrids of 2-thiohydantoins and rhodanine derivates. Other 2-thiohydantoin and rhodanine derivatives investigated by Tejchman et al. [2] demonstrated no activity against Gram-negative bacteria, but some of them were active against Gram-positive ones. Thiohydantoins reported by Camargo de Carvalho et al. [3] were more effective against Gram-positive bacteria, as well. The possible antibacterial mechanism of the hybrids of 2-thiohydantoin and rhodanine derivates reported by Tejchman et al. [2] suggests “their interaction with molecular targets located on the surface of the cell membrane”. Cho et al. [53] indicate for another possible mechanism of thiohydantoins acting as inhibitors of c-di-AMP synthase. This enzyme is often found in Gram-positive bacteria playing the role of a second messenger [53]. The possible mechanism for explaining the reduction of our compounds activity and the lack of bactericidal effect could be a decreased permeation of the hybrids into bacterial cells because of the large size of their molecules and location on the surface of the cell envelope. However, this hypothesis requires confirmation.

#### 2.3.2. Antibacterial Activity under Blue Light Irradiation

Upon blue light irradiation of the compounds, singlet oxygen and a stream of free radicals generation could affect the antibacterial effect. On the bases of the conducted research concerning direct detection of singlet oxygen phosphorescence, all studied quinolone derivatives generate singlet oxygen upon excitation with 420 nm, however with different efficiency. Therefore, we used blue light irradiation of microtitrate plates with tested compounds and bacteria to investigate an antibacterial effect under blue light conditions (Table 6, Table 7 and Table 8).

A decrease of MIC values was noticed in case of **4e** for three bacterial strains: *B. subtilis, K. pneumoniae*, and *P. aeruginosa* in comparison to the dark group. In case of **5f** after the irradiation with blue light, the Gram-negative strains did not show a decrease in MIC values relative to the dark. However, in contrast, Gram-positive bacteria revealed a significant decrease in MIC values under the influence of blue light. **5e** was inactive both in the darkness and upon irradiation. **4c** was inactive at all strains except of *P. aeruginosa* after the exposition to blue light. In case of **5c** we observed a decrease of MIC values for *P. aeruginosa* and *B. subtilis* while for *E. faecalis*, and *S. aureus* there was no difference between the MIC values in the dark and in the blue light group. After irradiation *K. pneumonia* became sensitive to this compound. **4d** was activated by blue light which caused the MIC to drop to low values (approx. 3.91–62.5 µg/mL) for all the bacterial strains. In case of **4f,** light activated this compound, which could be seen for *S. aureus* and *B. subtilis* strains. The light activation and its impact on all bacterial strains was observed for **4b**. **5b** did not affect Gram-negative strains. On the other hand, it had a stronger effect on Gram-positive when exposed to blue light. Concluding, Table 8 shows that the tested compounds had a weak to moderate light-enhanced bacteriostatic activity. In contrast, they had no bactericidal activity as indicated by MBC/MIC ratios that equal 8 or more (8–64).

In case of compounds **4a**, **4c**, and **5e**, the MIC and MBC values were not determined for most of the tested bacterial strains due to the relatively low solubility of these compounds and potential aggregation.

On the bases of the biophysical studies described above, it was concluded that the blue light activation of most of the tested compounds led to the release of singlet oxygen. A decrease of the MIC values was observed after the irradiation of the microtitrate plates with blue light in comparison to the dark group. Generally, the effect of enhancement of the antibacterial activity of most of the tested new compounds is observed upon blue light irradiation (**4b**, **4d**, **4f**, **5c**, **5f**). For most of the compounds the higher sensitivity is noticed for Gram-positive bacteria and *P. aeruginosa*. Such an observation is in line with previously reported [34]. We suspect that the activity of the studied compounds irradiated with blue light might be a result of their binding to the cell membranes of the bacteria. Such a situation is reported, i.e., for merocyanine [54].

Blue light leads to the excitation of intracellular photosensitizing compounds, i.e., porphyrins, which act as photosensitizers in a classical PDT. The absorption of a photon leads to the transfer of energy and the final generation of highly cytotoxic reactive oxygen species, particularly singlet oxygen [55].

In current study a synergism of novel compounds and blue light treatment (BLT) was observed, leading to the increased susceptibility of *P. aeruginosa* to antimicrobial agents (**4e** and **5c** were bactericidal because of MBC/MIC = 1, Table 8). As previously reported, *P. aeruginosa* isolates pretreated with sublethal BLT showed increased sensitivity to antibiotics such as gentamycin, ceftazidime, and meropenem. MIC values were 2-, 8-, and even 64-fold lower after sequential blue light irradiation and antibiotic administration [56]. Similar effect was reported by Ronqui et al. [57]. They showed the synergistic antimicrobial effects of photodynamic therapy with methylene blue and ciprofloxacin against *S. aureus* and *E. coli*. 

#### 2.3.3. Structure and Microbiological Activity Relationship

Analyzing the quinolone antibiotics reported, the differences in the chemical structure of these compounds and the related different range of action of these drugs are observed. The quinolones can be modified at a few positions to optimize activity, pharmacokinetics, and toxicity. Improvements in activity and antiresistant properties of these derivatives are still possible and required [58,59,60]. As an example of the structure–activity relationship considering such organic systems we can look to the work by Al-Amiery et al. [10]. They investigated the antibacterial activity of Schiff’s bases containing 2-quinolone core and observed that the linkage of 2-quinolone and a moiety including the heterocyclic nitrogen atom led to a better antimicrobial effect in comparison with the linkage with a moiety containing the heterocyclic oxygen atom. Bolokatti et al. [16] reported the antimicrobial activity of other quinolone derivatives, namely a series of 3-(2-(4-(6,7-disubstituted-benzo[d]thiazol-2-yl)phenylamino)acetyl)−4-hydroxy-1-methyl/phenyl quinolin-2(1*H*)-one derivatives directed against Gram-positive and Gram-negative bacteria. The activity of the examined compounds was low in case of Gram-positive bacteria, but moderate towards Gram-negative ones. The compounds inhibited the growth of *E. coli* and *P. aeruginosa* and the derivatives containing the phenyl group in N-1 position revealed higher activity than the derivatives containing the methyl group. 

In our antimicrobial investigations, we observed that the acetate group –CH_2_-COOH located in the thiohydantoin ring might influence the reactivity of compounds. **5e** having this substituent was microbiologically inactive in contrast to **4e** without this substituent showing moderate bacteriostatic activity. However, other compounds with this group at N3 atom of 2-thiohydantoin ring were significantly active (**5c** towards Gram-positive bacteria) or at least showed moderate microbiological activity (**5f**). Therefore, it is difficult to conclude unambiguously about the influence of this acetate group on the antibacterial activity. Presence of –Br substituent in the 2-quinolone ring did not guarantee the high microbiological activity even after blue light irradiation. Both **5c** and **4c** have one bromide group at the same position, however, only **5c** was active (towards Gram-positive bacteria in the dark and towards most of the bacteria strains upon irradiation), whereas **4c** showed very limited activity only towards *P. aeruginosa* and only after irradiation. The high bacteriostatic activity of **5c** might be a result of the combination of the presence of acetate group in the 2-thiohydantoin ring and bromide substituent in the 2-quinolone ring. A certain rule may be noticed considering the influence of the methyl group –CH_3_ in the 2-quinolone ring. In cases of **4e**, **4d**, and **4f** with this substituent (independently on its position), but in the concurrent absence of the acetate group in 2-thiohydantoin ring, the distinct bacteriostatic activity was observed. The activity of these compounds was enhanced by blue light. The simultaneous presence of these methyl and acetate groups did not guarantee the bacteriostatic activity of the compound (e.g., **5e** was inactive, whereas **5f** was moderate active).

## 3. Materials and Methods

### 3.1. Chemistry

Chemicals for the synthesis of 2-thiohydantoin and 2-thiohydantoin-3-acetic acid derivatives were obtained from Sigma-Aldrich and used without subsequent purification. Melting points of all compounds (uncorrected) were determined on the Boetius apparatus. The IR spectra were recorded with Jasco FT IR-670 Plus spectrophotometer in the KBr disk. The NMR spectra were obtained in DMSO-d_6_ at 300 K, on the Bruker 600 MHz Avance III spectrometer operating at 600.21 MHz (1H). The chemical shifts (ppm) were referenced to lock out the signal of the solvent and *J* was expressed in Hz. Mass spectrometry analysis was made using AmaZon ETD mass spectrometer (Bruker Daltonics, Bremen, Germany) equipped with standard electrospray ion source and ion trap analyzer. Samples were dissolved in the mixture of methanol and chloroform (1:1, *v*/*v*) with addition of 0.1% formic acid (*v*/*v*). Usually, concentration of the sample in solution did not exceed 1 nM. Samples were introduced into a mass spectrometer using a syringe pump with a flow rate of 2 mL/min. The following settings of the mass spectrometer were used: scan range 100–700 m/z, ion polarity: positive, heated capillary temperature: 280 °C, capillary voltage: −4500 V, ion trap was set to accumulate ca. 300,000 ions for a single cycle. Fragmentation was done after manual ion selection with an isolation window set to 4 Da. Low energy collision induced dissociation (low energy CID) was used as a fragmentation technique with the collision energy of 1 eV. Typically for ion traps, fragmentation cut-off was set to 27% of the nominal m/z ratio of selected parent ion. Fragmentation spectra were acquired for about 20 s (equal to ca. 20 spectra) and accumulated.

#### 3.1.1. General Procedure for the Condensation of 1-acetyl-2-thiohydantoin and 2-thiohydantoin-3-acetic Acid with 2-chloro-3-quinolinecarboxaldehyde Derivatives

**Figure 3:**
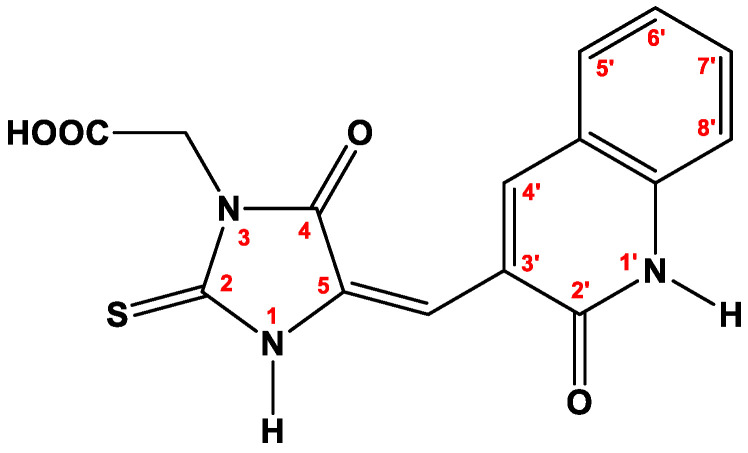
Atom-numbering scheme of the tested compounds.

The synthesis of 1-acetyl-2-thiohydantoin and 2-thiohydantoin-3-acetic acid, used at subsequent synthetic stages, was published earlier [2]. To a solution of 1-acetyl-2-thiohydantoin (0.791 g, 5 mmol) (1) (Scheme 1, in Results and Discussion) or of 2-thiohydantoin-3-acetic acid (0.871 g, 5 mmol) (2) (Scheme 2, in Results and Discussion) in glacial acetic acid (50 mL), anhydrous sodium acetate was added (1.231 g, 15 mmol, 3 eq.). Then the appropriate 2-chloro-3-quinolincarbaldehyde (5 mmol) was added to the resulting solution. The reaction mixture was heated within 5–6 h under reflux. In the next step the content of the flask was poured into cold water (100 cm^3^) and the obtained product was filtered off. Finally, the product was crystallized from either glacial acetic acid or acetic anhydride. As a result of the condensation reaction two series of compounds were obtained (Figure 3, Scheme 1 and Scheme 2). 

#### 3.1.2. Spectrometry and Spectroscopy

/**4a**/ 5-(((quinolin-2-(1*H*)-one)-3-yl)-methylene)-2-sulfanylideneimidazolidin-4-one, m.p. > 300 °C, yield; ES MS: [M-1]^−^-270, IR cm^−1^, (1 mg/400 mg KBr): 1750.08 (C=O, C-4), 1647.88 (C=O, C-2′), 1610.27 (C=C, exo.), 1221.68 (C=S), ^1^H NMR: 12.41 (s, 1H, N3), 12.26 (s, 1H, N1), 12.24 (s, 1H, N1′), 8.47 (s, 1H, C4′), 7.71 (d, 1H, *J* = 8.1 Hz, C5′ Ar-H), 7.58 (t, 1H, C7′ Ar-H), 7.37 (d, 1H, *J* = 8.0 Hz, C8′ Ar-H), 7.26 (t, 1H, C6′ Ar-H), 6.53 (s,1H, =CH-).

/**4b**/ 5-(((6-fluoro-quinolin-2-(1*H*)-one)-3-yl)-methylene)-2-sulfanylideneimidazolidin-4-one, m.p. > 300 °C, yield; ES MS: [M-1]^−^-288, IR cm^−1^, (1 mg/400 mg KBr): 1742.37 (C=O, C-4), 1644.02 (C=O, c-2′), 1615.09 (C=C, exo.), 1228.43, C=S), ^1^H NMR: 12.47 (s, 1H, N-3), 12.33 (s, 1H, N-1), 12.23 (s, 1H, N-1′), 8.41 (s,1H, C4′), 7.52 (dd, 1H, *J* = 8.9 Hz, *J* = 2.9 Hz, Ar-H), 7.48 (td, 1H, *J* = 8.9 Hz, *J* = 2.9 Hz, Ar-H), 7.38 (dd, 1H, *J* = 8.9 Hz, *J* = 4.5 Hz, Ar-H), 6.50 (s, 1H, =CH-).

/**4c**/ 5-(((6-bromo-quinolin-2-(1*H*)-one)-3-yl)-methylene)-2-sulfanylideneimidazolidin-4-one, m.p. > 300 °C, yield; ES MS: [M-1]^−^-348, 350, IR cm^−1^, (1 mg/400 mg KBr): 1725.98 (C=O, C-4), 1650.77 (C=O, C-2′), 1600.63 (C=C, exo.), 1222.65 (C=S), ^1^H NMR: 12.48 (s, 1H, N-3), 12.34 (s, 1H, N-1), 12.15 (s, 1H, N-1′), 8.40 (s,1H, C4′), 7.91 (d, 1H, *J* = 2.1 Hz, Ar-H), 7.70 (dd, 1H, *J* = 8.7 Hz, *J* = 2.1 Hz, Ar-H), 7.29 (d, 1H, *J* = 8.7 Hz, Ar-H), 6.50 (s, 1H, =CH-).

/**4d**/ 5-(((6-methyl-quinolin-2-(1*H*)-one)-3-yl)-methylene)-2-sulfanylideneimidazolidin-4-one, m.p. > 300 °C, yield; ES MS: [M-1]^−^-284, IR cm^−1^, (1 mg/400 mg KBr): 1725.98 (C=O, C-4), 1650.77 (C=O, C-2′), 1226.50 (C=S), ^1^H NMR: 12.41 (s, 1H, N-3), 12.25 (s, 1H, N-1), 12.22 (s, 1H, N-1′), 8.39 (s, 1H, C4′), 7.49 (s, 1H, Ar-H), 7.40 (d, 1H, *J* = 7.9 Hz, Ar-H), 7.26 (d, 1H, *J* = 7.9 Hz, Ar-H), 6.50 (s, 1H, =CH-), 2.35 (s, 3H, CH_3_).

/**4e**/ 5-(((7-methyl-quinolin-2-(1*H*)-one)-3-yl)-methylene)-2-sulfanylideneimidazolidin-4-one, m.p. > 300 °C, yield; ES MS: [M-1]^−^-284, IR cm^−1^, (1 mg/400 mg KBr): 1725.98 (C=O, C-4), 1638.23 (C=O, C-2′), 1617.02 (C=C, exo.), 1224.47 (C=S), ^1^H NMR: 12.38 (s, 1H, N-3), 12.21 (s, 1H, N-1), 12.19 (s, 1H, N-1′), 8.37 (s,1H, C4′), 7.57 (d, 1H, *J* = 7.9 Hz, Ar-H), 7.12 (s, 1H, Ar-H), 7.07 (d, 1H, *J* = 7.9 Hz, Ar-H), 6.49 (s, 1H, =CH-), 2.38 (s, 3H, CH_3_).

/**4f**/ 5-(((8-methyl-quinolin-2-(1*H*)-one)-3-yl)-methylene)-2-sulfanylideneimidazolidin-4-one, m.p. > 300 °C, yield; ES MS: [M-1]^−^-284, IR cm^−1^, (1 mg/400 mg KBr): 1726.34 (C=O, C-4), 1635.34 (C=O, C-2′), 1599.66 (C=C, exo.), 1227.47 (C=S), ^1^H NMR: 12.43 (s, 1H, N-3), 12.25 (s, 1H, N-1), 11.98 (s, 1H, N-1′), 8.49 (s, 1H, C4′), 7.58 (d, 1H, *J* = 7.0 Hz, Ar-H), 7.42 (d, 1H, *J* = 7.0 Hz, Ar-H), 7.17 (t, 1H, *J* = 7.0 Hz, Ar-H), 6.49 (s, 1H, =CH-), 2.45 (s, 3H, CH_3_).

/**5a**/ 2-(5-((quinolin-2-(1*H*)-one)-3-yl)-methylene)-(4-oxo-2-sulfanylideneimidazolidin-3-yl) acetic acid, m.p. > 300 °C, yield; ES MS: [M-1]^−^-328, IR cm^−1^, (1 mg/400 mg KBr): 1751.05 (C=O, -COOH), 1730.80 (C=O, C-4), 1635,34 (C=O, C-2′), 1607.38 (C=C, exo.), 1222.65 (C=S), ^1^H NMR: 12.32–12.00 (m, 3H, -COOH, N-1, N-1′), 8.68 (s, 1H, C4′), 7.71 (d, 1H, *J* = 8.0 Hz, Ar-H), 7.55 (t, 1H, *J* = 8.0 Hz, Ar-H), 7.35 (d, 1H, *J* = 8.0 Hz, Ar-H), 7.25 (t, 1H, *J* = 8.0 Hz, Ar-H), 6.65 (s, 1H,=CH-), 4.48 (s, 2H, -CH_2_-).

/**5b**/ 2-(5-((6-fluoro-quinolin-2-(1*H*)-one)-3-yl)-methylene)-(4-oxo-2-sulfanylideneimidazolidin-3-yl) acetic acid, m.p. > 300 °C, yield; ES MS: [M-1]^−^-346, IR cm^−1^, (1 mg/400 mg KBr): 1753.37 (C=O, -COOH), 1728.87 (C=O, C-4), 1640.16 (C=O, C-2′), 1615.09 (C=C, exo.), 1219.36 (C=S), ^1^H NMR: 12.90–12.00 (m, 3H, -COOH, N-1, N-1′), 8.49 (s, 1H, C4′), 7.53 (dd, 1H, *J* = 9.0 Hz, *J* = 2.9 Hz, Ar-H), 7.48 (dt, 1H, *J* = 9.0 Hz, *J* = 2.9 Hz, Ar-H), 7.39 (dd, 1H, *J* = 9.0 Hz, *J* = 4.8, Ar-H), 6.68 (s, 1H,=CH-), 4.49 (s, 2H, -CH_2_-).

/**5c**/ 2-(5-((6-bromo-quinolin-2-(1*H*)-one)-3-yl)-methylene)-(4-oxo-2-sulfanylideneimidazolidin-3-yl) acetic acid, m.p. > 300 °C, yield; ES MS: [M-1]^−^-406, 408, IR cm^−1^, (1 mg/400 mg KBr): 1774.19 (C=O, -COOH), 1726.94 (C=O, C-4), 1637.27 (C=O, C-2′), 1598.70 (C=C, exo.), 1221.68 (C=S), ^1^H NMR: 12.70–12.30 (m, 3H, -COOH, N-1, N-1′), 8.47 (s, 1H, C4′), 7.53 (d, 1H, *J* = 2.2 Hz, Ar-H), 7.72 (dd, 1H, *J* = 8.7 Hz, *J* = 2.2 Hz, Ar-H), 7.30 (d, 1H, *J* = 8.7 Hz, Ar-H), 6.68 (s, 1H,=CH-), 4.50 (s, 2H, -CH_2_-).

/**5d**/ 2-(5-((6-methyl-quinolin-2-(1*H*)-one)-3-yl)-methylene)-(4-oxo-2-sulfanylideneimidazolidin-3-yl) acetic acid, m.p. > 300 °C, yield; ES MS: [M-1]^−^-342, IR cm^−1^, (1 mg/400 mg KBr): 1763.07 (C=O, -COOH), 1717.30 (C=O, C-4), 1643.05 (C=O, C-2′), 1615.09 (C=C, exo.), 1221.68 (C=S), ^1^H NMR: 12.86 (brs, 1H, -COOH), 12.66 (s, 1H, N-1), 12.26 (s, 1H, N-1′), 8.43 (s, 1H, C4′), 7.48 (s, 1H, Ar-H), 7.41 (d, 1H, *J* = 8.0 Hz, Ar-H), 7.27 (d, 1H, *J* = 8.0 Hz, Ar-H), 6.68 (s, 1H,=CH-), 4.50 (s, 2H, -CH_2_-), 2.36 (s, 1H, CH_3_).

/**5e**/ 2-(5-((7-methyl-quinolin-2-(1*H*)-one)-3-yl)-methylene)-(4-oxo-2-sulfanylideneimidazolidin-3-yl) acetic acid, m.p. > 300 °C, yield; ES MS: [M-1]^−^-342, IR cm^−1^, (1 mg/400 mg KBr): 1752.09 (C=O, -COOH), 1725.01 (C=O, C-4), 1641.13 (C=O, C-2′), 1603.52 (C=C, exo.), 1221.68 (C=S), ^1^H NMR: 13.20 (brs, 1H, -COOH), 12.65 (s, 1H, N-1), 12.27 (s, 1H, N-1′), 8.49 (s, 1H, C4′), 7.62 (d, 1H, *J* = 8.5 Hz, Ar-H), 7.17 (s, 1H, Ar-H), 7.12 (d, 1H, *J* = 8.5 Hz, Ar-H), 6.70 (s, 1H,=CH-), 4.51 (s, 2H, -CH_2_-), 2.42 (s, 1H, CH_3_).

/**5f**/ 2-(5-((8-methyl-quinolin-2-(1*H*)-one)-3-yl)-methylene)-(4-oxo-2-sulfanylideneimidazolidin-3-yl) acetic acid, m.p. > 300 °C, yield; ES MS: [M-1]^−^-342, IR cm^−1^, (1 mg/400 mg KBr): 1753.39 (C=O, -COOH), 1728.87 (C=O, C-4), 1658.48 (C=O, C-2′), 1595.84 (C=C, exo.), 1220.72 (C=S), ^1^H NMR: 13.25 (brs, 1H, -COOH), 12.67 (s, 1H, N-1), 11.46 (s, 1H, N-1′), 8.56 (s, 1H, C4′), 7.60 (d, 1H, *J* = 7.5 Hz, Ar-H), 7.45 (d, 1H, *J* = 7.5 Hz, Ar-H), 7.20 (t, 1H, *J* = 7.5 Hz, Ar-H), 6.76 (s, 1H,=CH-), 4.53 (s, 2H, -CH_2_-), 2.47 (s, 1H, CH_3_).

The UV–vis absorption spectra were recorded recorded in DMSO: ethanol (1:1, *v*/*v*), at ambient temperature, in 1 cm quartz cuvette using Hewlett-Packard—HP 8452A Diode Array Spectrophotometer (Agilent Technologies Inc. Markham, Ontario, Canada). The emission spectra of the examined compounds were also recorded in DMSO: ethanol (1:1, *v*/*v*), at ambient temperature, in 1 cm quartz cuvette Hellma using spectrofluorometer Perkin-Elmer LS50B. Samples were excited at 420 nm and the emission was measured within a range of 440–540 nm. The absorption values of the samples at the excitation wavelength (420 nm) were in the range 0.08–0.11. 

### 3.2. Direct Detection of Singlet Oxygen (^1^Δ_g_, ^1^O_2_*) Phosphorescence at 1270 nm

The following solvents were used for the detection of singlet oxygen phosphorescence: DMSO—HPLC grade, Avantor Performance Materials Poland (Gliwice, Poland); ethanol—HPLC grade, Merck KGaA (Darmstadt, Germany); perinaphthenone Merck KGaA (Darmstadt, Germany).

Before measurements, samples were dissolved in DMSO and ethanol (1:1, *v*/*v*), placed in a quartz fluorescence cuvette (QA-1000; Hellma, Mullheim, Germany) and excited with 420 nm laser pulse. Excitation light was generated by an integrated nanosecond DSS Nd:YAG laser system equipped with a narrow bandwidth optical parametric oscillator (NT242-1k-SH/SFG; Ekspla, Vilnius, Lithuania). Relative yield of singlet oxygen (^1^O_2_*, ^1^Δ_g_), generation upon excitation with 420 nm was determined by a comparative method, employing perinaphthenone (1-phenalenone, PN) as a standard [41]. In these experiments, initial intensities of singlet oxygen phosphorescence in the studied sample and in standard excited with laser pulses were measured at increasing laser energies. Absorbance of standard and studied sample were adjusted to ~0.10 at excitation wavelength 420 nm. 

The near-infrared phosphorescence of generated singlet oxygen was measured perpendicularly to the excitation beam in a photon-counting mode using a thermoelectric cooled NIR PMT module (H10330-45; Hamamatsu, Japan) equipped with a 1100 nm cut-off filter and a dichroic narrow band filter NBP of the spectral range 1150–1355 nm (NDC Infrared Engineering Ltd., Bates Road, Maldon, Essex, UK). Data analysis, including first-order luminescence decay fitted by the Levenberg–Marquardt algorithm, was performed by custom-written software. 

### 3.3. Antibacterial Activity Assay In Vitro

#### 3.3.1. Bacterial Strains

The antibacterial activity of each compound was evaluated using six bacterial strains: three strains of Gram-positive (*Staphylococcus aureus* ATCC 292313, *Bacillus subtilis* ATCC 6633, and *Enterococcus faecalis* ATCC 29212) and three strains of Gram-negative (*Escherichia coli* ATCC 25922, *Pseudomonas aeruginosa* ATCC 27853, and *Klebsiella pneumoniae* ATCC 700603) bacteria. The bacterial strains were purchased from American Type Culture Collection (ATCC) (Manassas, VA, USA). 

#### 3.3.2. Antibacterial Assay Preparation 

The species of bacteria were grown at 37 °C for 24 h, in Miller’s LB Broth (A&A Biotechnology, Gdańsk, Poland). The bacterial growth was harvested using 5 mL of sterile saline water, its absorbance was adjusted at 580 nm and diluted to achieve viable cell count of 10^6^ CFU/mL using densitometer (DEN-1 Mc Farland Densitometer). A stock solution of each tested compound was prepared in DMSO (10 mg/mL). The 2-fold serial dilutions were made ranging from 1000 μg/mL to 3.91 μg/mL using LB Broth (Miller) in 96-well microplates. Higher concentrations of these compounds were not achieved, because of the lack of solubility. 10 μL of the bacterial suspension was inoculated into each well and 200 μL of the solution prepared from the above-mentioned stock solution and filled with LB Broth (Miller) was added. The final concentration of DMSO in each well was less than 5% (*v*/*v*) and preliminary analyses with 5% (*v*/*v*) DMSO did not inhibit the growth of the test bacteria (data not shown). A sterility control well and a growth control well were also studied for each strain. The investigations were conducted for two experimental groups. The first group of microtitrate plates was kept in the dark as a control and the second one was irradiated with a blue light (Flood Light LED RGB 50W Premium LUX (Warsaw, Poland), 446.37 nm, FWHM = 18 nm, 34 mW/cm^2^, 1.5 h) (See Figure S3 in Supplementary Materials). All the microtiter plates were incubated at 37 °C within 20 h. The MIC (Minimum Inhibitory Concentration) values were determined spectrophotometrically (λ = 580 nm; Epoch, BioTech, Winooski, VT, USA). The concentration, that inhibited bacterial growth completely (the first clear well), was taken as the MIC value. MIC values were determined at least in triplicate and repeated to confirm activity. Minimal bactericidal concentration (MBC) was determined by subculturing 100 μL of the microbial culture from each well that showed through growth inhibition, from the last positive one and from the growth control onto the Petri dish with agar. The plates were incubated at 37 °C for 20 h and the MBC was defined as the lowest concentration of the compounds without growth of microorganisms. Antibacterial activity of the tested compounds was compared with ciprofloxacin as a standard antibiotic (Table 4). Each experiment was repeated in triplicate. Representative data is presented.

## 4. Conclusions

On the bases of our preliminary results in vitro, the novel synthesized hybrid compounds consisting of a 2-thiohydantoin core and a 2-quinolone ring show bacteriostatic activity. Blue light activation of the tested compounds leads to singlet oxygen generation with moderate yield as we demonstrated by direct detection of ^1^O_2_* (^1^Δ_g_) phosphorescence at 1270 nm. The irradiation with blue light enhances the bacteriostatic effect of most tested compounds. Gram-positive bacteria show a greater sensitivity to the novel synthesized 2-quinolone derivatives compared to Gram-negative bacteria. The enhancement of the bacteriostatic effect of the tested compounds is observed in *P. aeruginosa* after blue light irradiation. The obtained results indicate the further direction of structural modifications of these derivatives in order to enhance their lipophilicity and antibacterial properties.

## Figures and Tables

**Table 1:** Relative efficiency of singlet oxygen generation by studied samples calculated against the standard.

Sample	Relative Efficiency of ^1^O_2_ (^1^Δ_g_) Generation (%)
PN (standard)	100
**4c**	12.43 (±0.02)
**4a**	10.90 (±1.07)
**4b**	10.23 (±0.93)
**4f**	9.81 (±0.24)
**4d**	8.54 (±0.23)
**5c**	7.98 (±0.56)
**4e**	7.71 (±0.98)
**5a**	5.91 (±0.66)
**5f**	5.77 (±1.14)
**5d**	5.66 (±0.15)
**5b**	4.93 (±0.22)
**5e**	3.93 (±2.10)

**Table 2:** MIC (μg/mL), MBC (μg/mL) of **4a**, **4b**, **4c**, **4d**, and **4e** towards reference Gram-positive and Gram-negative bacterial strains in dark conditions.

Microorganism	4a		4b		4c		4d		4e	
	MIC (μg/mL)	MBC (μg/mL)	MIC (μg/mL)	MBC (μg/mL)	MIC (μg/mL)	MBC (μg/mL)	MIC (μg/mL)	MBC (μg/mL)	MIC (μg/mL)	MBC (μg/mL)
*E. coli*	nd	nd	nd	nd	nd	nd	nd	nd	500	nd
*P. aeruginosa*	nd	nd	nd	nd	nd	nd	nd	nd	1000	nd
*K. pneumoniae*	nd	nd	nd	nd	nd	nd	250	nd	31.25	1000
*E. faecalis*	nd	nd	7.82	250	nd	nd	7.82	250	7.82	500
*S. aureus*	nd	nd	7.82	125	nd	nd	125	nd	31.25	1000
*B. subtilis*	nd	nd	nd	nd	nd	nd	7.82	250	62.5	nd

nd—not determined.

**Table 3:** MIC (μg/mL), MBC (μg/mL) of **4f**, **5b**, **5c**, **5e**, and **5f** towards reference Gram-positive and Gram-negative bacterial strains in dark conditions.

Microorganism	4f		5b		5c		5e		5f	
	MIC (μg/mL)	MBC (μg/mL)	MIC (μg/mL)	MBC (μg/mL)	MIC (μg/mL)	MBC (μg/mL)	MIC (μg/mL)	MBC (μg/mL)	MIC (μg/mL)	MBC (μg/mL)
*E. coli*	62.5	1000	nd	nd	nd	nd	nd	nd	500	nd
*P. aeruginosa*	31.25	500	nd	nd	nd	nd	nd	nd	250	nd
*K. pneumoniae*	62.5	1000	nd	nd	nd	nd	nd	nd	250	nd
*E. faecalis*	62.5	1000	250	nd	62.5	1000	nd	nd	250	nd
*S. aureus*	31.25	500	250	nd	62.5	1000	nd	nd	250	nd
*B. subtilis*	31.25	500	250	nd	125	nd	nd	nd	62.5	1000

nd—not determined.

**Table 4:** MBC to MIC ratios calculated for the compounds showing an antibacterial activity in the dark conditions.

Microorganism	4b	4d	4e	4f	5b	5c	5f
*E. coli*	-	-	-	16	-	-	-
*P. aeruginosa*	-	-	-	16	-	-	-
*K. pneumoniae*	-	-	32	16	-	-	-
*E. faecalis*	32	32	64	16	-	16	-
*S. aureus*	16	-	32	16	-	16	-
*B. subtilis*	-	32	-	16	-	-	16

**Table 5:** MIC (μg/mL), MBC (μg/mL), and MBC/MIC ratio of ciprofloxacin towards reference Gram-positive and Gram-negative bacterial strains.

Microorganism	Ciprofloxacin		
	MIC (μg/mL)	MBC (μg/mL)	MBC/MIC Ratio
*E. coli* ATCC 25922	0.24	0.48	2
*P. aeruginosa* ATCC 27853	0.26	0.31	1.19
*K. pneumoniae* ATCC 700603	7.81	7.81	1
*E. faecalis* ATCC 29212	1	1	1
*S. aureus* ATCC 29213	7.81	7.81	1
*B. subtilis* ATCC 6633	0.98	0.98	1

**Table 6:** MIC (μg/mL), MBC (μg/mL) of **4a**, **4b**, **4c**, **4d**, and **4e** towards reference Gram-positive and Gram-negative bacterial strains upon blue light irradiation (see text for details).

Microorganism	4a		4b		4c		4d		4e	
	MIC (μg/mL)	MBC (μg/mL)	MIC (μg/mL)	MBC (μg/mL)	MIC (μg/mL)	MBC (μg/mL)	MIC (μg/mL)	MBC (μg/mL)	MIC (μg/mL)	MBC (μg/mL)
*E. coli*	nd	nd	125	1000	nd	nd	7.82	250	500	nd
*P. aeruginosa*	nd	nd	7.82	250	3.91	125	3.91	125	3.91	3.91
*K. pneumoniae*	nd	nd	62.5	1000	nd	nd	62.5	1000	15.63	500
*E. faecalis*	nd	nd	7.82	250	nd	nd	7.82	250	15.63	500
*S. aureus*	nd	nd	7.82	125	nd	nd	3.91	125	31.25	1000
*B. subtilis*	nd	nd	7.82	125	nd	nd	3.91	125	3.91	250

nd—not determined.

**Table 7:** MIC (μg/mL), MBC (μg/mL) of **4f**, **5b**, **5c**, **5e,** and **5f** towards reference Gram-positive and Gram-negative bacterial strains upon blue light irradiation (see text for details).

Microorganism	4f		5b		5c		5e		5f	
	MIC (μg/mL)	MBC (μg/mL)	MIC (μg/mL)	MBC (μg/mL)	MIC (μg/mL)	MBC (μg/mL)	MIC (μg/mL)	MBC (μg/mL)	MIC (μg/mL)	MBC (μg/mL)
*E. coli*	62.5	1000	nd	nd	nd	nd	nd	nd	500	nd
*P. aeruginosa*	7.82	125	nd	nd	3.91	3.91	nd	nd	7.82	250
*K. pneumoniae*	62.5	1000	nd	nd	62.5	1000	nd	nd	250	nd
*E. faecalis*	62.5	1000	31.25	1000	62.5	1000	nd	nd	62.5	nd
*S. aureus*	3.91	250	125	1000	62.5	1000	nd	nd	15.63	500
*B. subtilis*	3.91	250	125	1000	3.91	250	nd	nd	3.91	250

nd—not determined.

**Table 8:** The MBC to MIC ratios calculated for the compounds showing an antibacterial activity upon blue light irradiation.

Microorganism	4b	4d	4e	4f	5b	5c	5f
*E. coli*	8	32	-	16	-	-	-
*P. aeruginosa*	32	32	1	16	-	1	-
*K. pneumoniae*	16	16	32	16	-	16	-
*E. faecalis*	32	32	32	16	32	16	-
*S. aureus*	16	32	32	64	8	16	32
*B. subtilis*	16	32	64	64	8	64	64

## Data Availability

All data generated or analyzed during this study are included in this published article. Moreover, the datasets used and/or analyzed during the current study are available from the corresponding author on reasonable request.

## Supplementary Materials

Figure S1 (A–L): The absorption spectra of the examined compounds **4a**–**5f**, recorded in DMSO: ethanol (1:1, *v*/*v*) at ambient temperature, in the wavelength range 300–550 nm; spectra normalized to 1 at maximum of the main absorption band; Figure S2 (A–L): The emission spectra of the examined compounds **4a**–**5f**, recorded in DMSO: ethanol (1:1, *v*/*v*) at ambient temperature, in the wavelength range 440–540 nm. Λex = 420 nm; Figure S3: The blue light region characteristics of the lamp Flood Light LED RGB 50W Premium LUX (Warsaw, Poland), applied in the antimicrobial experiments (see text for details). The characteristics of the lamp was performed using spectroradiometer GL Spectis 5.0 Touch (GL Optic).

## Sample Availability

Samples of the investigated and described compounds are available from the authors.
