# Supplementary material for: Preliminary Studies of Antimicrobial Activity of New Synthesized Hybrids of 2-Thiohydantoin and 2-Quinolone Derivatives Activated with Blue Light
Source: Molecules. 2022 Feb 5;27(3):1069. doi: 10.3390/molecules27031069 (PMC8839260; doi:10.3390/molecules27031069)
Supplement: Supplementary file 1 [file molecules-27-01069-s001.zip › molecules-1417678-supplementary.pdf]

molecules

MDPI

Supplementary Materials

Preliminary Studies of Antimicrobial Activity of New Synthesized Hybrids of 2-Thiohydantoin and 2-Quinolone Derivatives Activated with Blue Light

Agnieszka Kania [1,*], Waldemar Tejchman [1], Anna M. Pawlak [2], Krystian Mokrzyński [2], Bartosz Różanowski [1], Bogdan M. Musielak [3] and Magdalena Greczek-Stachura [1]

1. Absorption spectra

A

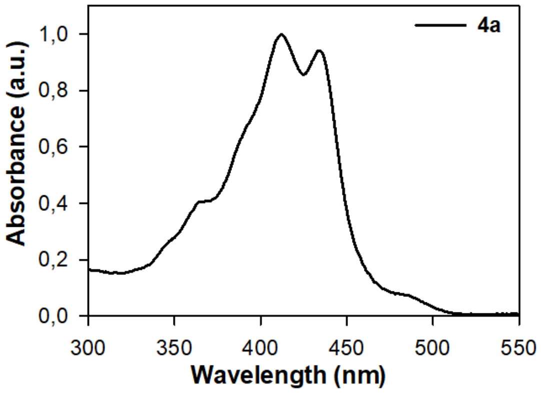

B

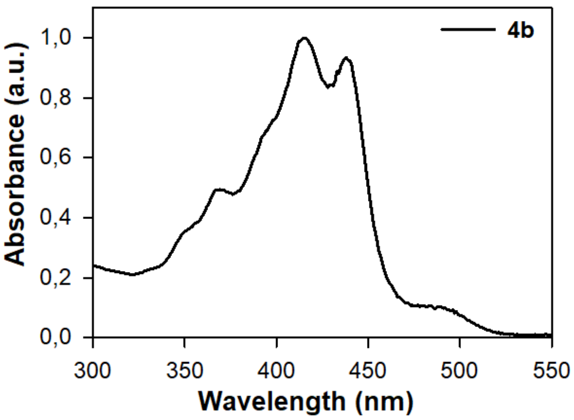

C

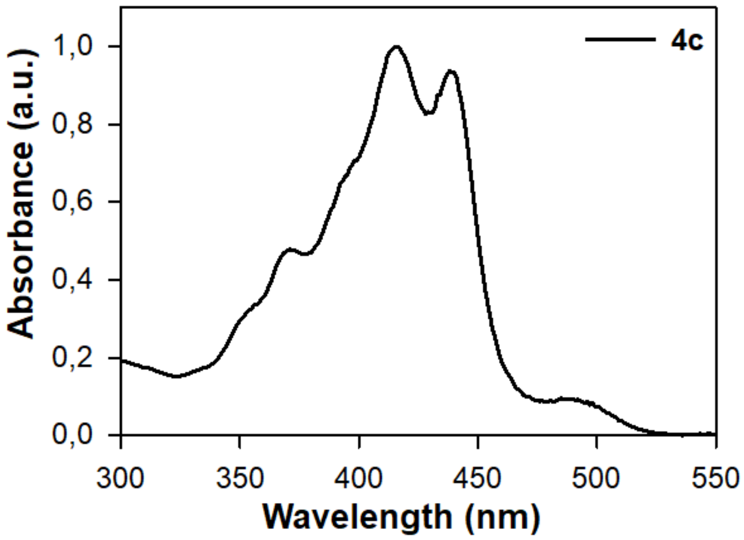
4c
Absorbance (a.u.)
1,0
0,8
0,6
0,4
0,2
0,0
300
350
400
450
500
550
Wavelength (nm)

D

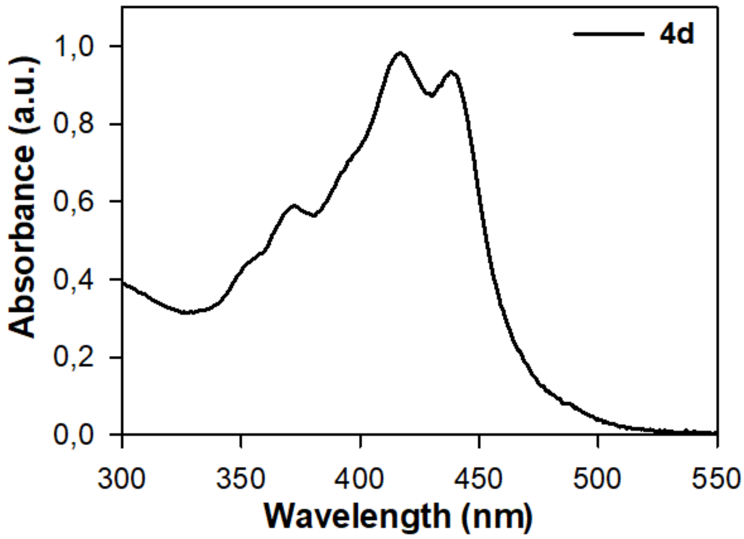
4d
Absorbance (a.u.)
1,0
0,8
0,6
0,4
0,2
0,0
300
350
400
450
500
550
Wavelength (nm)

E

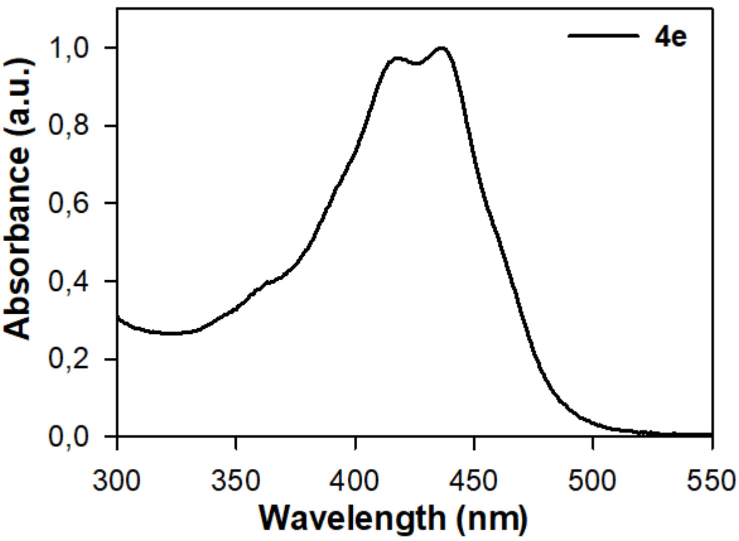
4e
Absorbance (a.u.)
1,0
0,8
0,6
0,4
0,2
0,0
300
350
400
450
500
550
Wavelength (nm)

F

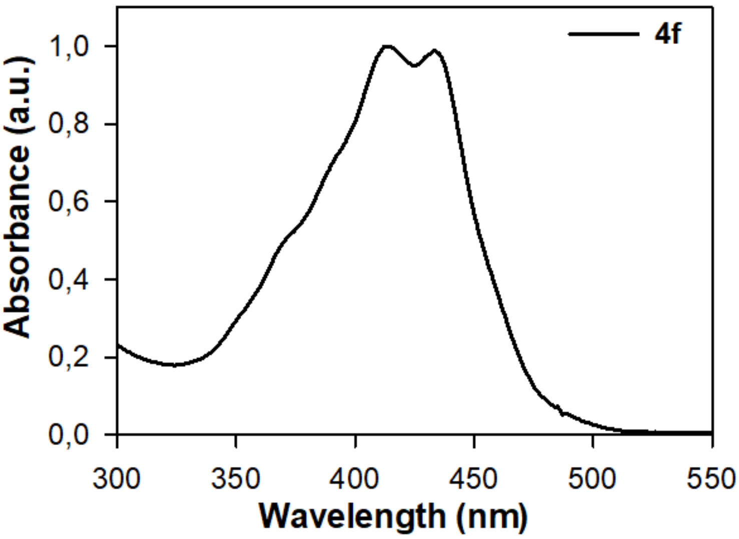
4f
Absorbance (a.u.)
1,0
0,8
0,6
0,4
0,2
0,0
300
350
400
450
500
550
Wavelength (nm)

G

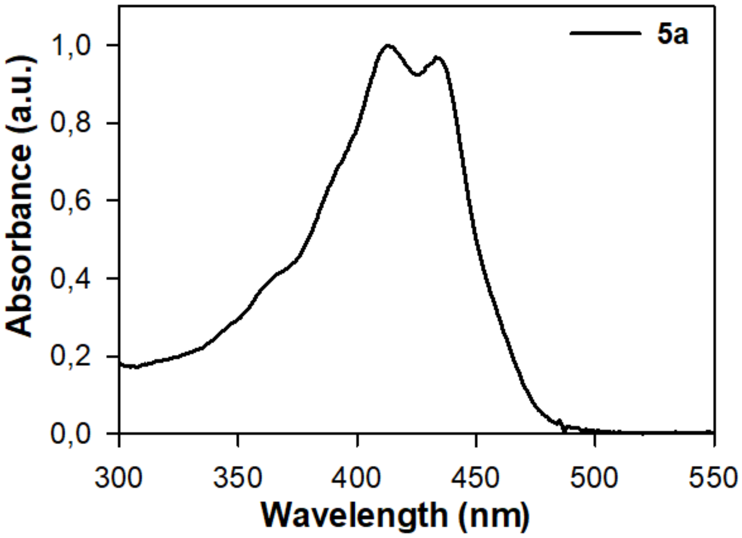
5a
Absorbance (a.u.)
1,0
0,8
0,6
0,4
0,2
0,0
300
350
400
450
500
550
Wavelength (nm)

H

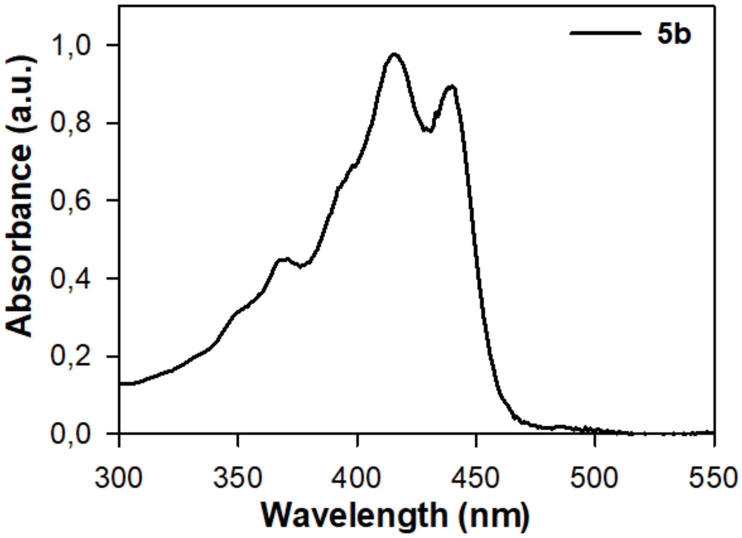
5b
Absorbance (a.u.)
1,0
0,8
0,6
0,4
0,2
0,0
300
350
400
450
500
550
Wavelength (nm)

I

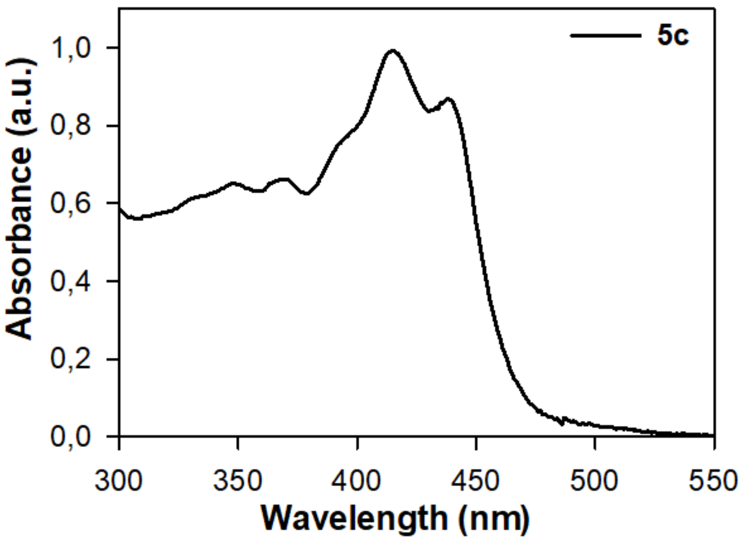
5c
Absorbance (a.u.)
1,0
0,8
0,6
0,4
0,2
0,0
300
350
400
450
500
550
Wavelength (nm)

J

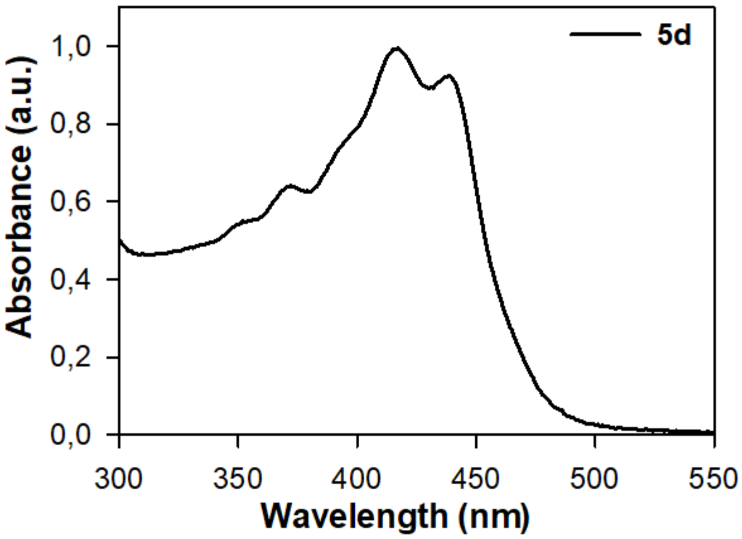
5d
Absorbance (a.u.)
1,0
0,8
0,6
0,4
0,2
0,0
300
350
400
450
500
550
Wavelength (nm)

K

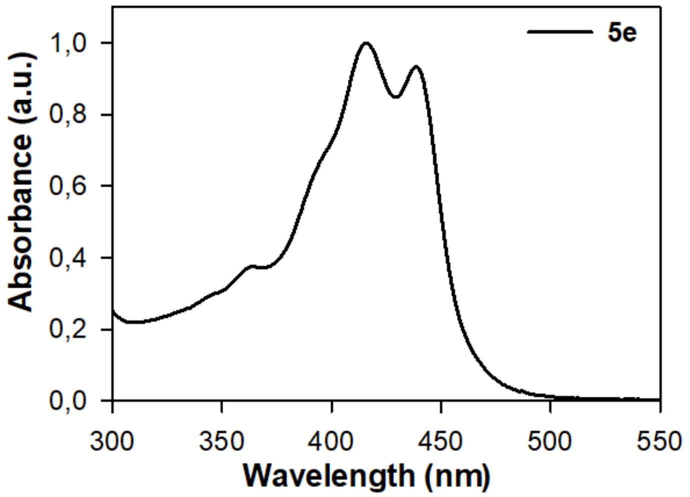

L

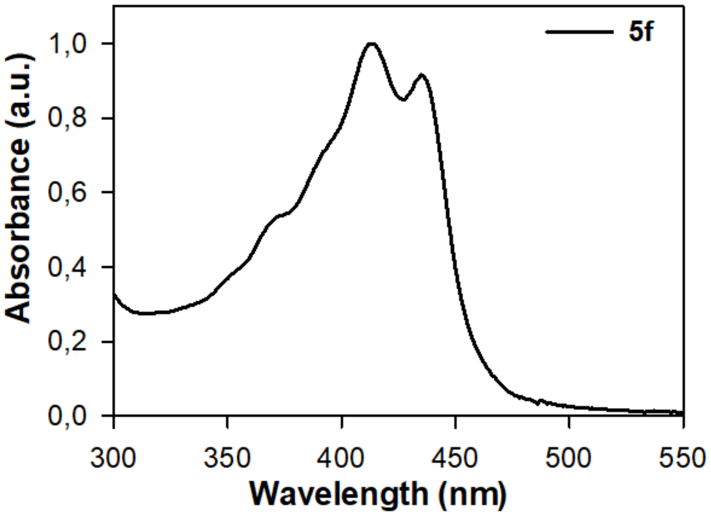

Figure S1. (A-L). The absorption spectra of the examined compounds **4a-5f**, recorded in DMSO: ethanol (1:1, v/v) at ambient temperature, in the wavelength range 300-550 nm; spectra normalized to 1 at maximum of the main absorption band.

2. Emission spectra

A

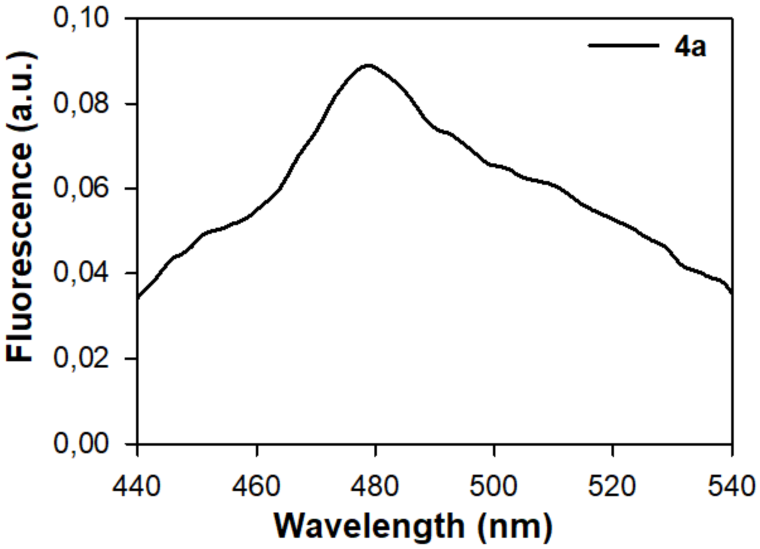

B

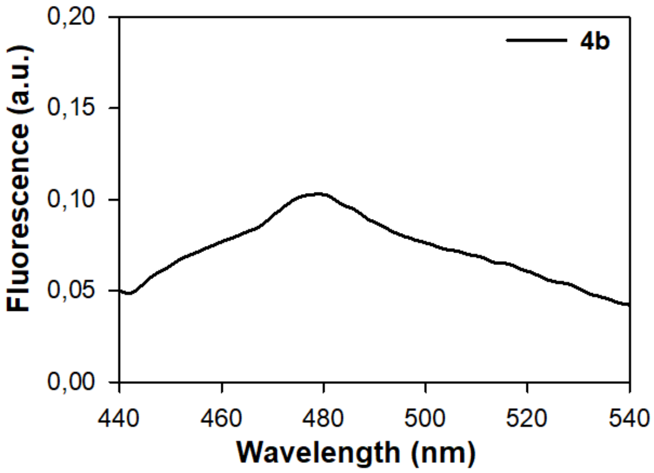

C

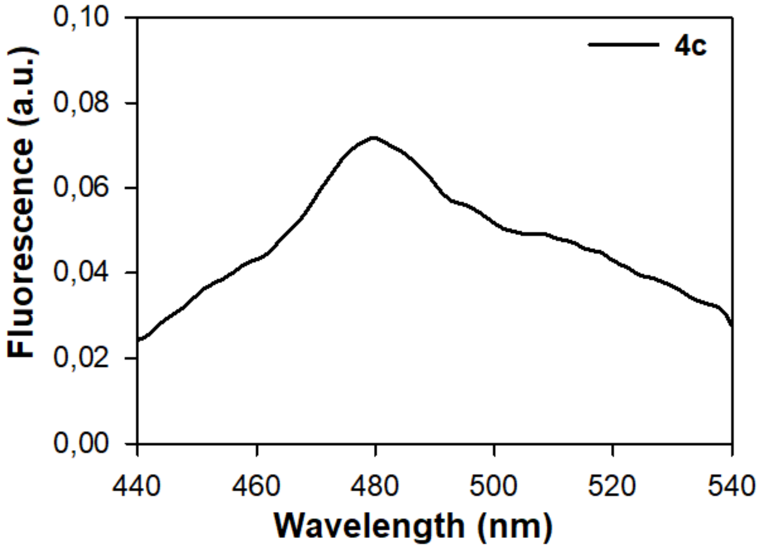

4c
Fluorescence (a.u.)
0,10
0,08
0,06
0,04
0,02
0,00
440
460
480
500
520
540
Wavelength (nm)

D

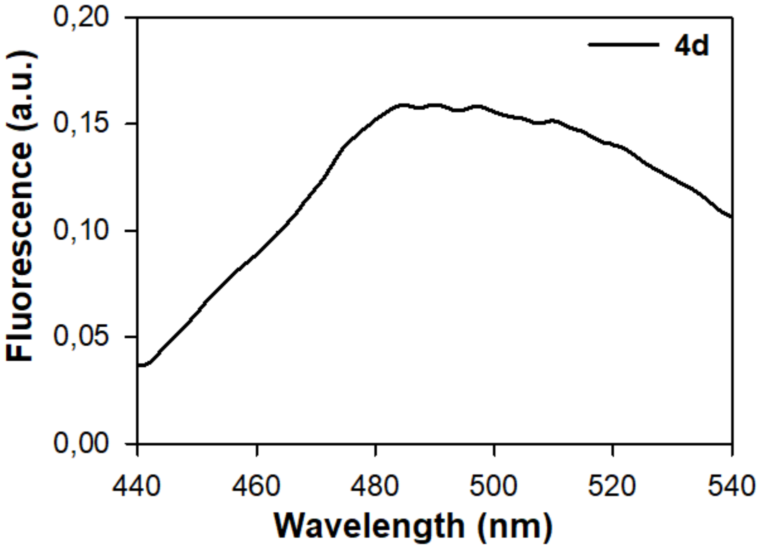

4d
Fluorescence (a.u.)
0,20
0,15
0,10
0,05
0,00
440
460
480
500
520
540
Wavelength (nm)

E

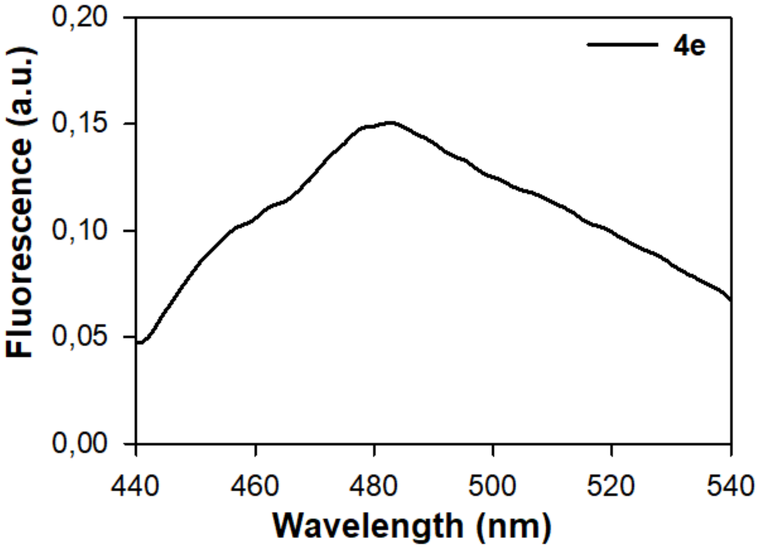

4e
Fluorescence (a.u.)
0,20
0,15
0,10
0,05
0,00
440
460
480
500
520
540
Wavelength (nm)

F

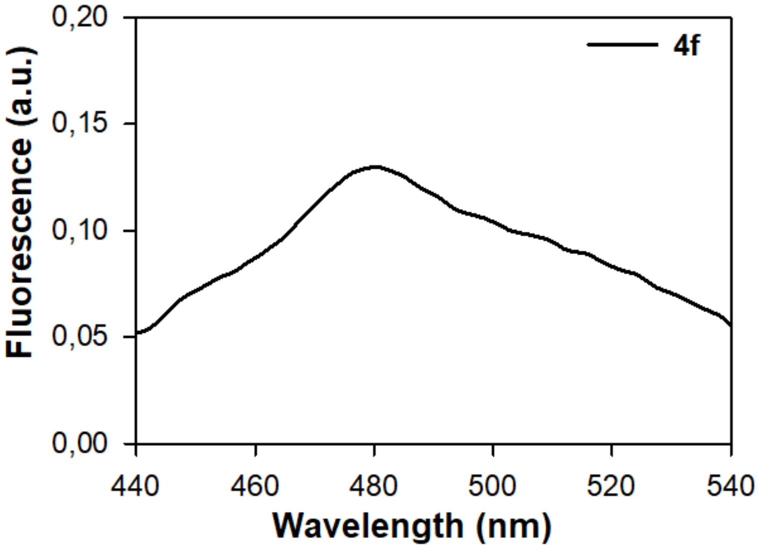

4f
Fluorescence (a.u.)
0,20
0,15
0,10
0,05
0,00
440
460
480
500
520
540
Wavelength (nm)

G

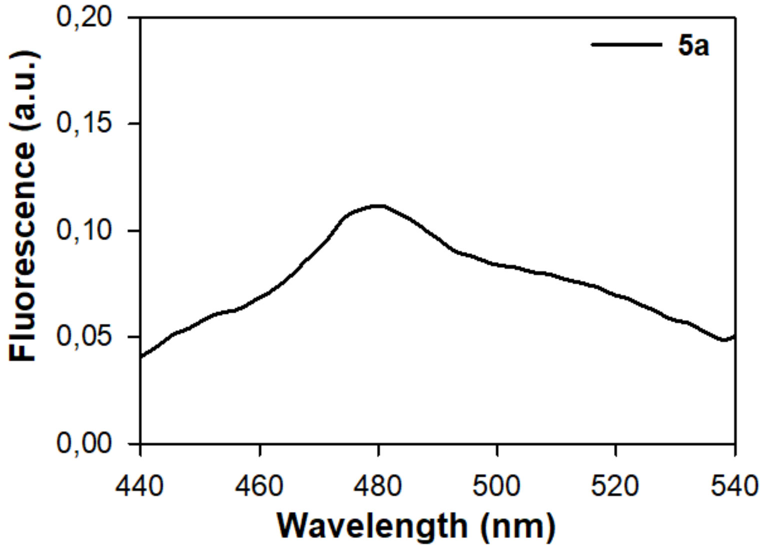
5a
0,20
0,15
0,10
0,05
0,00
Fluorescence (a.u.)
440
460
480
500
520
540
Wavelength (nm)

H

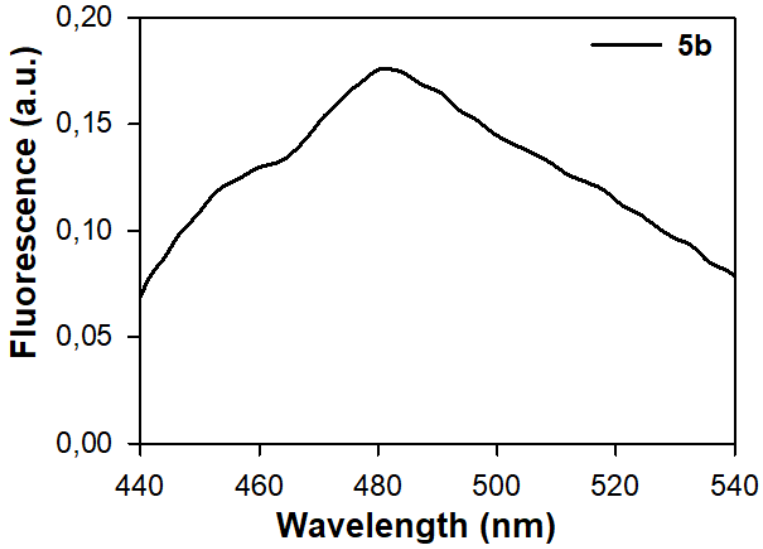
5b
0,20
0,15
0,10
0,05
0,00
Fluorescence (a.u.)
440
460
480
500
520
540
Wavelength (nm)

I

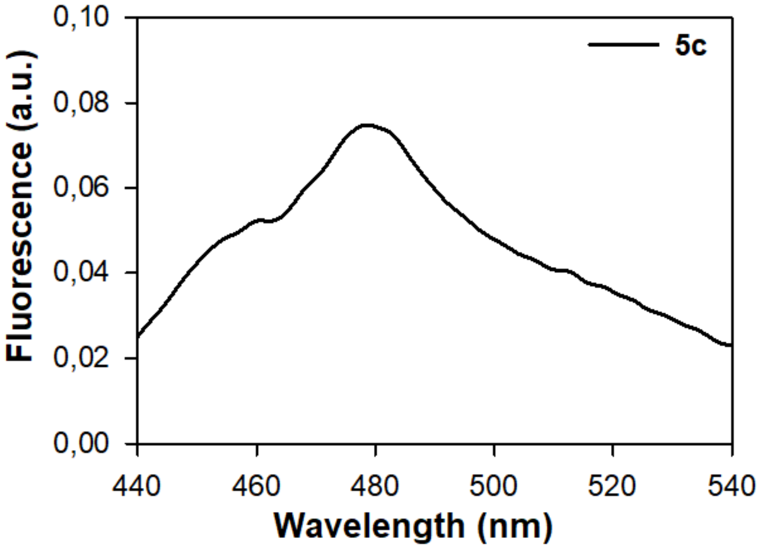
0,10
0,08
0,06
0,04
0,02
0,00
Fluorescence (a.u.)
5c
440
460
480
500
520
540
Wavelength (nm)

J

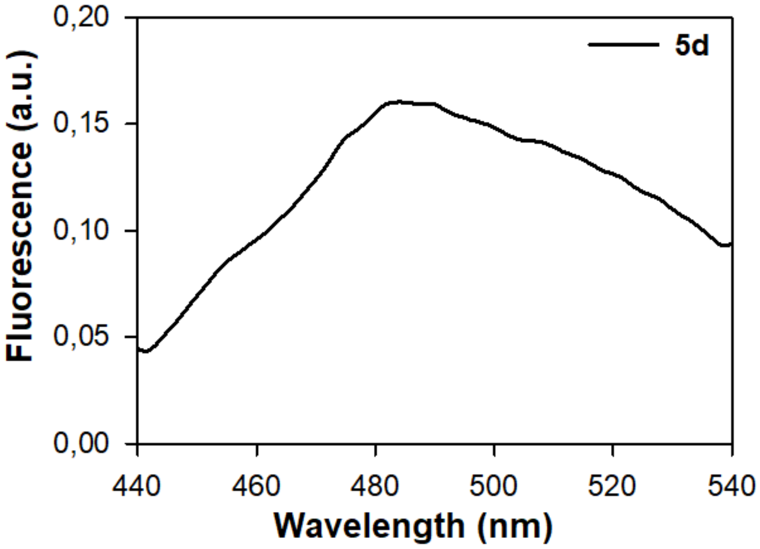
0,20
0,15
0,10
0,05
0,00
Fluorescence (a.u.)
5d
440
460
480
500
520
540
Wavelength (nm)

K

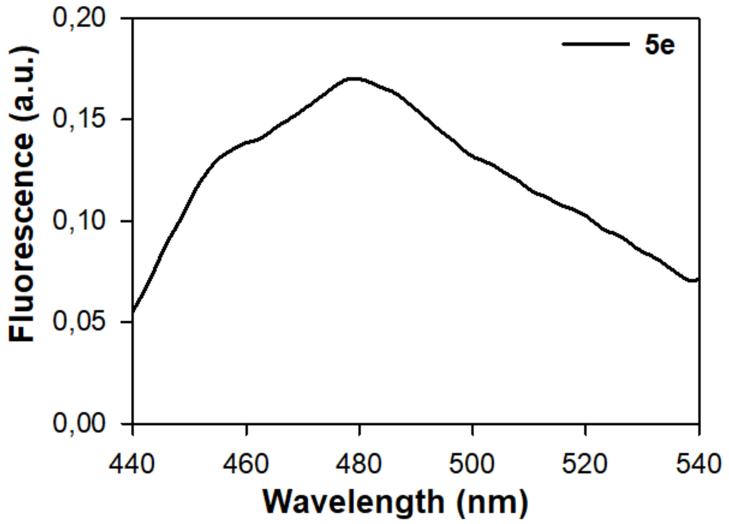

L

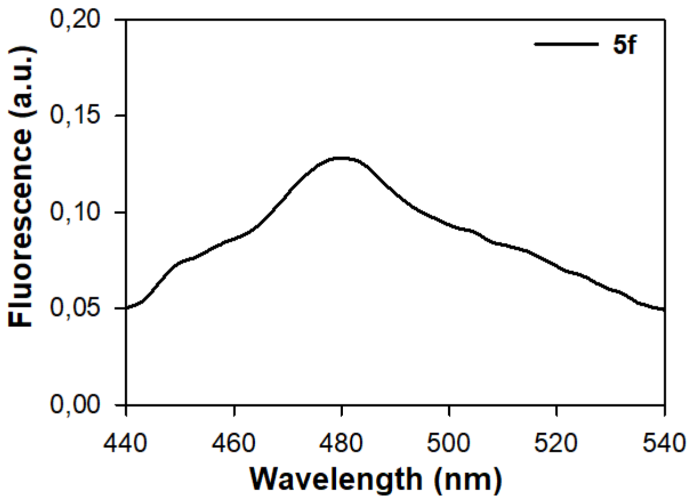

Figure S2. (A-L). The emission spectra of the examined compounds **4a-5f**, recorded in DMSO:—ethanol (1:1, v/v) at ambient temperature, in the wavelength range 440-540 nm. Λ_{ex} = 420 nm.

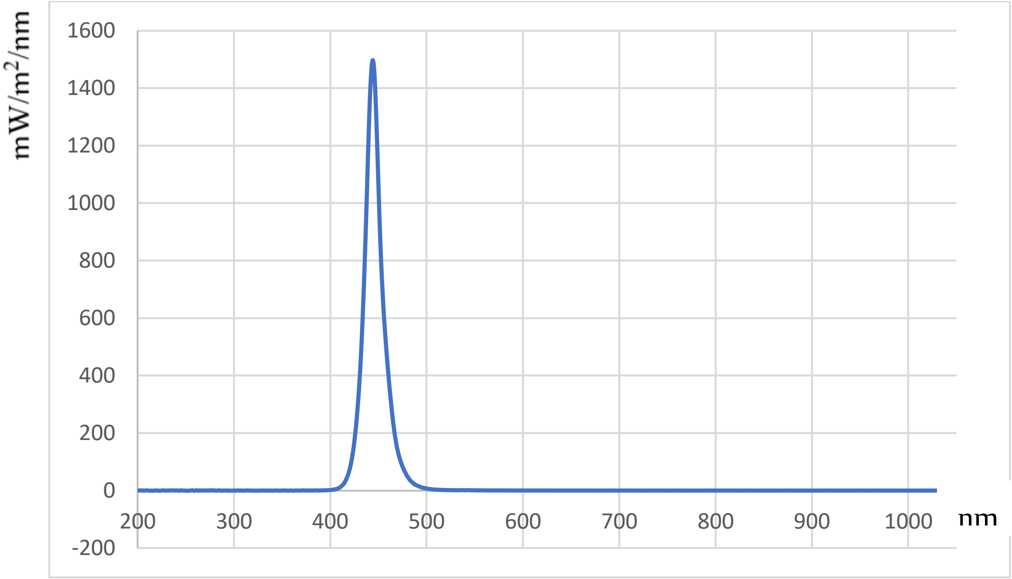

Figure S3. The blue light region characteristics of the lamp Flood Light LED RGB 50W Premium LUX (Warsaw, Poland), applied in the antimicrobial experiments (see text for details). The characteristics of the lamp was performed using spectroradiometer GL Spectis 5.0 Touch (GL Optic).
